# Synergistic modulation of capacitive-diffusive charge storage kinetics in an Ni-SnS_2_@SnO_2_ nanocomposite for advanced micro-supercapacitor applications

**DOI:** 10.1039/d6ra06761a

**Published:** 2026-09-25

**Authors:** Ravindra Kumar, Diksha Sharma, Geetika Patel, Gurupada Maity, Ashish Kumar Keshari

**Affiliations:** a Department of Applied Physics, School of Vocational Studies and Applied Sciences, Gautam Buddha University Greater Noida 201312 India ashishk@gbu.ac.in; b Department of Physics, School of Basic Sciences, Galgotias University Gautam Buddha Nagar-203201 India gurupada.phy@gmail.com; c Department of Chemistry & Environmental Sustainability, School of Science, IILM University Gautam Buddha Nagar-201312 Greater Noida India

## Abstract

Metal sulfide/oxide nanocomposites are promising electrode materials for high-performance supercapacitors; however, the charge-storage kinetics of Ni-doped SnS_2_@SnO_2_ remain insufficiently explored. Moreover, previous studies on SnO_2_-based electrodes have mainly focused on enhancing capacitance, while the underlying charge-storage kinetics, particularly the distinction between capacitive and diffusion-controlled contributions, remain largely unexplored. Herein, an Ni-SnS_2_@SnO_2_ nanocomposite was synthesized by a simple, cost-effective one-step solvothermal method and characterized by XRD, FESEM, EDX, and HR-TEM. SnS_2_, SnS_2_@SnO_2_, and Ni-SnS_2_@SnO_2_ electrodes were evaluated in 1 M Na_2_SO_4_ using CV, GCD, and EIS in a three-electrode configuration. Structural analysis confirmed the formation of the SnS_2_@SnO_2_ heterostructure, while Ni incorporation induced lattice expansion and increased interlayer spacing, facilitating ion accessibility and rapid intercalation/deintercalation. At 0.5 mA cm^−2^, Ni-SnS_2_@SnO_2_ delivered an areal capacitance of 202.63 mF cm^−2^, superior to the 66.32 and 108.06 mF cm^−2^ delivered by SnO_2_ and SnS_2_@SnO_2_, respectively. Kinetic analysis revealed a mixed capacitive-diffusion-controlled mechanism arising from heterostructure formation and Ni doping, which enhanced charge transport and introduced additional redox-active sites. The electrode retained ∼85% of its capacitance after 5000 cycles and achieved 18.01 mW h cm^−2^ energy density at 199.98 mW cm^−2^ power density. A coulombic efficiency of 85.05–99.93% further demonstrates excellent reversibility and rate capability. These results highlight Ni-SnS_2_@SnO_2_ as a promising electrode for advanced micro-supercapacitors and next-generation energy storage systems.

## Abstract 2

Ni-doped SnS_2_@SnO_2_ nanocomposites tune capacitive-diffusive charge storage through enhanced ion transport and lattice expansion, delivering 202.63 mF cm^−2^ capacitance, 85% capacity retention, and 18.01 mW h cm^−2^ energy density.

## Graphical abstract

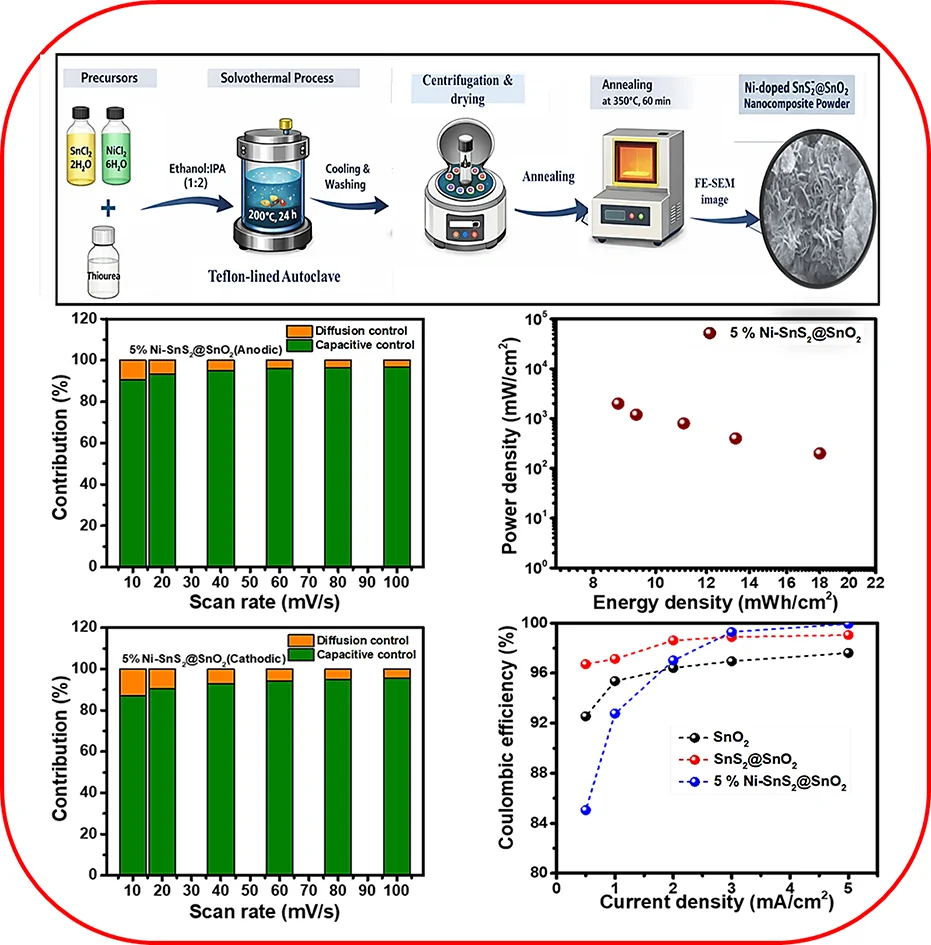

## 1 Introduction

The growing global energy demand has driven the rapid development of efficient energy storage systems compatible with renewable energy sources to fulfil the increased energy demands of electronic devices and electric vehicles. Supercapacitors (SCs), also known as electrochemical supercapacitors or ultracapacitors, have been globally recognized and utilized in diverse fields owing to their attractive features such as fast charging/discharging characteristics, long life-span, high power density, and safe operation in a wide temperature window.^1,2^ However, despite having these features, SCs suffer from low energy density.^3^ Moreover, the shape restriction of electrodes and performance degradation due to the involvement of metal collectors and binders during electrode fabrication restrict some specified applications of SCs, where the size plays a vital role. Therefore, the development of small supercapacitors, especially for portable electronics, has been recognized as a key direction for the advancement of next-generation energy storage devices. Micro-supercapacitors (MSCs) are the primary choice to fulfil advanced miniaturized energy demands due to their rapid frequency response, adequate power density, fast charge/discharge rate capabilities, and longevity.^2^ The key advantage of MSCs is their miniaturized architecture, allowing their seamless integration into sensors and wearable electronics without significantly adding weight to the bulk.^2,4,5^

As widely recognized, the electrochemical performance of MSCs highly relies on the electrode material, and consequently, the electrochemical performance of these devices can be significantly improved through the optimization of the intrinsic properties of the electrode materials and rational design of the electrode architecture.^6,7^ From a materials perspective, high electronic and ionic conductivities are crucial for minimizing capacitance losses, particularly under high scan rates and current densities. Therefore, electrode materials should exhibit excellent electrical conductivity to facilitate rapid electron transport while possessing an optimized, ion-accessible structure that enables efficient ion diffusion. From an electrode design perspective, electrodes should be integrated with metallic current collectors through mechanically and chemically robust interfaces to ensure efficient charge transport, long-term cycling stability, and high-rate performance. Furthermore, increasing the effective surface area of the electrodes enhances the number of electrochemically active sites available for charge storage, thereby leading to improved specific capacitance.^8,9^ In addition, the electrochemical performance of electrodes can be significantly enhanced through nanoengineering and surface modification, improving the energy density and accelerating electron/ion transport kinetics. Electrode materials with high porosity, excellent electrical conductivity, large surface area, and rapid charge-transfer kinetics have been developed and refined extensively for achieving superior electrochemical performance.^6^

Recently, the metal oxides of Sn, Co, Ti, and Mn have been explored as promising electrode materials for the fabrication of solid-state SC devices owing to their advantageous characteristics, like their physical and electrical properties. Various nanostructured metal oxides have been developed for electrochemical energy storage applications owing to their ability to improve the specific capacitance and long-term cycling stability.^10,11^ In particular, SnO_2_ stands out as a potential electrode material in energy storage applications due to its increased capacitance, low toxicity, ease of synthesis, cost-effectiveness, and high theoretical capacity and electron mobility.^10,11^ However, SnO_2_ electrode suffers due to volume variation during the electrochemical (charging/discharging) process that hinders the performance. However, the practical application of SnO_2_ electrodes is limited by their substantial volume expansion during electrochemical cycling, which generates structural discontinuities, deteriorates charge-transfer pathways, and ultimately leads to capacitance fading.^12,13^ Furthermore, their practical application remains constrained by their structural instability and comparatively low specific capacitance. Integrating two-dimensional materials such as SnS_2_ with SnO_2_ nanoparticles to form a nanocomposite is a viable strategy to mitigate the problems of structural instability and volume fluctuations by facilitating enhanced charge transport. To date, a variety of nanostructured SnO_2_/carbon and SnO_2_/SnS_2_ composites have been developed using diverse synthetic approaches for applications in supercapacitors, sensors, and solar cells.^14–18^ The synergistic SnO_2_/SnS_2_ composites have emerged as promising electrode materials for energy storage devices, offering enhanced electrochemical performance and excellent cycling stability.^19^ Many studies have demonstrated that SnO_2_/SnS_2_ composites synthesized *via* different routes exhibit significantly improved electrochemical performance and long-term cycling stability.^14,20,21^ In an earlier study, Prabukumar *et al.* fabricated SnO_2_ nanoparticles and an MoS_2_–SnO_2_ nanocomposite *via* a hydrothermal method. In this study, SnO_2_ nanoparticles delivered only a specific capacitance of 14 F g^−1^ at 1 A g^−1^ in a 2 M KOH electrolyte solution, while the MoS_2_–SnO_2_ nanocomposite delivered a specific capacitance of 61.6 F g^−1^ under the same parameters, which is 4.4 times higher than that delivered by bare SnO_2_. Also, the MoS_2_–SnO_2_ nanocomposite revealed a capacitance retention of 90% even after 1000 cycles at a current density of 1 A g^−1^.^14^ In another study, Ahmed *et al.* fabricated ACW and an SnO_2_–ACW hybrid composite *via* an impregnation method, followed by calcination. During electrochemical tests, ACW displayed only a specific capacitance of 0.122 F g^−1^ at an actual current of 1 mA in a 1 M Na_2_SO_4_ aqueous electrolyte solution, while the SnO_2_–ACW hybrid composite exhibited a very high specific capacitance of 30.46 F g^−1^ under the same parameters. Moreover, the SnO_2_–ACW hybrid composite electrode retained 108.9% of its initial capacitance even after 6000 consecutive charge–discharge cycles under the same parameters.^22^ The above-mentioned SnO_2_-based composites with various structures have limited practical applications due to their low surface area. Therefore, the development of high-performance powdered SnO_2_-based composite electrode materials through a simple, cost-effective, and scalable synthesis route is highly desirable. Moreover, transition metal (*i.e.* Ni)-doped Ni-SnS_2_@SnO_2_ nanocomposites as electrode materials for supercapacitors have not yet been investigated rigorously. Ni was selected as a dopant because of its ability to modify the electronic structure and electrical conductivity of Sn-based materials, while Ni-associated redox processes can additionally contribute to faradaic charge storage. Furthermore, Ni was selected because of its multiple oxidation states (Ni^2+^/Ni^3+^), high redox activity, and favorable electronic structure, which can introduce additional active sites, improve charge-transfer kinetics, and promote ion diffusion. Ni incorporation also induces lattice distortion and increases interlayer spacing, enhancing the electrolyte-ion accessibility. Moreover, Ni is relatively low-cost and chemically compatible with Sn-based materials, making it a suitable dopant for enhancing charge/ion transport in SnS_2_@SnO_2_. Also, previous studies on SnO_2_-based electrodes have focused mostly on improving the capacitance values;^14–16,22–27^ the fundamental charge-storage kinetics, especially, the distinction between capacitive and diffusion-controlled processes, remain largely unexplored.

In the present work, pure SnO_2_, SnS_2_@SnO_2_, and the Ni-SnS_2_@SnO_2_ nanocomposite were fabricated using a facile one-step solvothermal method and systematically characterized to explicate their structural and morphological properties. Further, the electrochemical performance of these samples was examined in detail. A comprehensive kinetic analysis was also employed to explain the fundamental charge-storage mechanism and establish kinetic engineering as a viable strategy for next-generation micro-supercapacitor electrodes.

## 2 Experimental section

### 2.1 Chemicals and materials for the synthesis of the Ni-SnS_2_@SnO_2_ nanocomposite

Tin(ii) chloride dihydrate (SnCl_2_·2H_2_O), nickel(ii) chloride hexahydrate (NiCl_2_·6H_2_O), and thioacetamide (C_2_H_5_NS) were used. All chemical reagents with a purity of ≥99.9% were purchased from Sigma-Aldrich and used as received without further purification.

### 2.2 Synthesis of SnO_2_, SnS_2_@SnO_2_, and the Ni-SnS_2_@SnO_2_ nanocomposite

SnO_2_, SnS_2_@SnO_2_, and Ni-SnS_2_@SnO_2_ powders were synthesized *via* a facile solvothermal method, followed by a subsequent annealing process (Fig. 1). The samples were obtained using a previously reported solvothermal method with some modifications.^27^

**Fig. 1:**
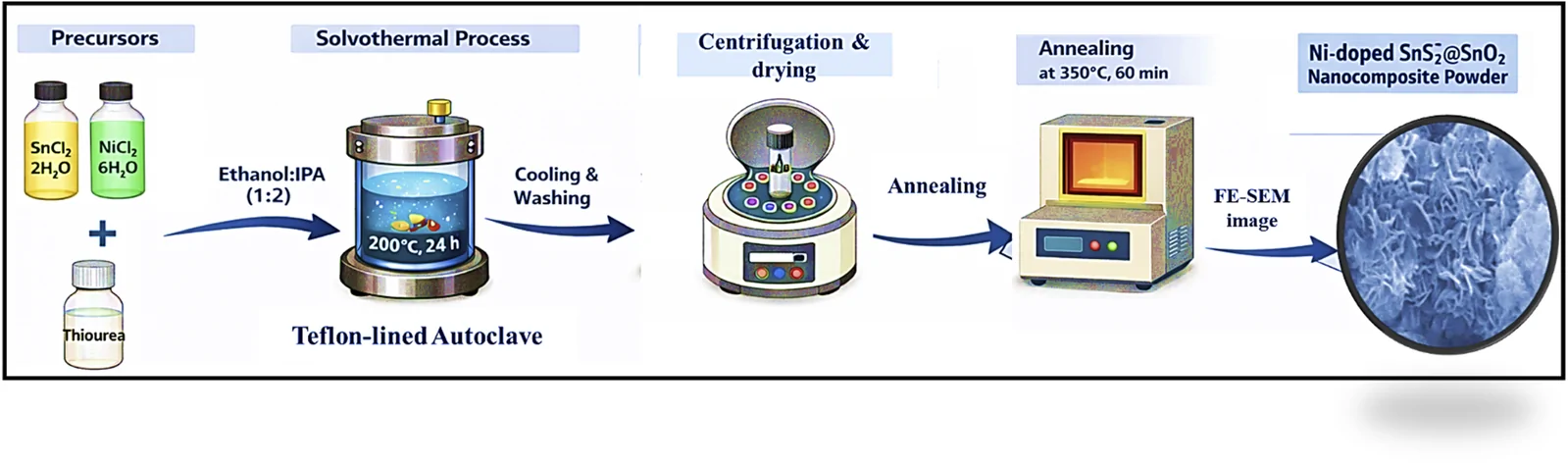
Schematic of the synthesis of the Ni-SnS_2_@SnO_2_ heterostructure nanocomposite.

#### 2.2.1 Preparation of SnO_2_

In a typical procedure, 50 mM SnCl_2_·2H_2_O was dissolved in 30 mL of a mixed solvent containing ethanol and isopropanol (volume ratio = 1 : 2) and stirred for 20 min to obtain a homogeneous solution. The reaction mixture was then transferred to a Teflon-lined stainless-steel autoclave, sealed securely, and subjected to solvothermal treatment at 200 °C for 24 h in an electric oven. After naturally cooling to room temperature, the obtained product was collected and centrifuged at 10 000–12 000 rpm for 10 min. The collected precipitate was repeatedly washed with deionized water and ethanol to remove any unreacted precursors and residual impurities, followed by vacuum drying at 120 °C overnight. Finally, the dried sample was annealed at 350 °C for 60 min to obtain the SnO_2_ sample.

#### 2.2.2 Preparation of SnS_2_@SnO_2_

For comparison, SnS_2_@SnO_2_ was synthesized using the same procedure as above. Additionally, after adding SnCl_2_·2H_2_O, followed by stirring, 100 mM C_2_H_5_NS (thioacetamide) was introduced into the solution under continuous stirring to form a uniform mixture. The obtained product was similarly washed, dried, and annealed under the same conditions to yield the SnS_2_@SnO_2_ composite.

#### 2.2.3 Preparation of Ni-SnS_2_@SnO_2_

For the preparation of Ni-SnS_2_@SnO_2_, SnCl_2_·2H_2_O (50 mM) was initially dissolved in 30 mL of an ethanol/isopropanol solvent mixture with a volume ratio of 1 : 2. The solution was magnetically stirred for 20 min to ensure complete dissolution and uniformity. NiCl_2_·6H_2_O (5 mM) was then added to the resulting solution and stirred for an additional 20 min to facilitate the homogeneous mixing of the metal precursors. Thereafter, 100 mM thioacetamide (C_2_H_5_NS) was gradually added under continuous stirring, and the mixture was continuously stirred until a uniform precursor solution was obtained. After that, the procedure same as above was followed to obtain the Ni-SnS_2_@SnO_2_ heterostructure nanocomposite. Herein, 5% Ni incorporation was selected as a representative doping level to investigate the effect of Ni on the structural and electrochemical properties of SnS_2_@SnO_2_, rather than as an optimized concentration.

### 2.3 Material characterization methods

The morphology and surface features of the as-synthesized nanostructures were examined using field-emission scanning electron microscopy (FESEM, JSM-7600F Plus, JEOL, Tokyo, Japan). The elemental composition of the samples was analysed by energy-dispersive X-ray spectroscopy (EDX) attached to the FESEM system. The microstructure and lattice characteristics were further investigated using transmission electron microscopy (TEM) and high-resolution transmission electron microscopy (HRTEM) (JEM-F200, JEOL, Japan) operated at an accelerating voltage of 200 kV from a field emission gun. The crystal structure and phase purity of the synthesized materials were characterized by X-ray diffraction (XRD) using a Bruker D8 Discover diffractometer (Karlsruhe, Germany) in *θ*–2*θ* geometry with Cu Kα radiation (*λ* = 0.15406 nm). The surface elemental composition and chemical states of the samples were analysed by X-ray photoelectron spectroscopy (XPS, ESCALAB 250, Thermo Fisher Scientific, UK) employing a monochromatic Al Kα X-ray source (*hν* = 1486.6 eV).

### 2.4 Electrochemical measurements

The as-prepared active material, carbon black, *N*-methyl-2-pyrrlodone (NMP) solvent, poly-vinylidene fluoride (PVDF) binder, and carbon cloth (CC) substrate were used to fabricate the electrodes. The preparation of functioning electrodes from the obtained samples is summarized as follows. In order to prepare homogeneous and viscous slurries from the obtained powders, an appropriate portion of the as-prepared active material, carbon black and poly-vinylidene fluoride (PVDF) was combined at a weight ratio of 7 : 2 : 1 (0.7 mg of the active material, 0.2 mg of carbon black and 0.1 mg of the PVDF binder) and dispersed in the *N*-methyl-2-pyrrlodone (NMP) solvent. Thereafter, each slurry was drop-casted over a carbon cloth (CC) substrate with a geometrical area of 1 × 1 cm^2^, which was well cleaned prior to use. Afterward, the electrodes were dried at about 120 °C in vacuum overnight. Finally, the electrodes were acquired, and the mass loading of the active materials on the CC was calculated to be 2.7 mg cm^−2^.

The electrochemical capacitive performances of the prepared electrodes were tested in a 1 M Na_2_SO_4_ aqueous electrolyte solution using an Autolab PGSTAT302N instrument (Metrohm B.V., Utrecht, the Netherlands) controlled by the Nova (version 2.10) software with a three-electrode system at room temperature. During the test, a platinum (Pt) rod and silver/silver chloride (Ag/AgCl) were employed as the counter electrode and reference electrode, respectively. Cyclic voltammetry (CV) was recorded in the potential window of 0.0 V to 0.8 V (*vs.* Ag/AgCl) at a sweep rate of 10–100 mV s^−1^. Galvanostatic charge–discharge (GCD) measurements were carried out over a potential window of 0.0 to 0.8 V (*vs.* Ag/AgCl) at current densities ranging from 0.5 to 5.0 mA cm^−2^. Electrochemical impedance spectroscopy (EIS) measurements were performed by applying an AC voltage with an amplitude of 10 mV over a frequency range of 0.01 Hz to 10^5^ Hz.

For the CV analysis, the specific capacitance was calculated using the following formula:1
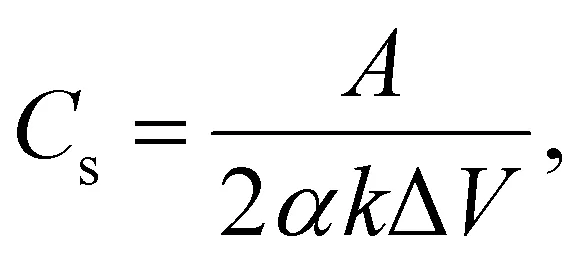
where *C*_s_ is the specific capacitance (F cm^−2^), and *A*, *α*, *k*, and Δ*V* are the area (CV curve), area of the active electrode material, sweeping rate, and potential window, respectively.

For the GCD analysis, the specific capacitance was calculated using the following formula:2
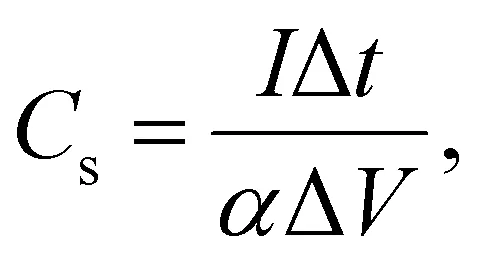
where *C*_s_ is the specific capacitance (F cm^−2^), 
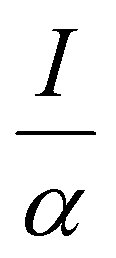
 is the current density (mA cm^−2^), Δ*t* is the discharging time (s), and Δ*V* the potential window (V).

Further, the energy density (*E*) in Wh cm^−2^ and the power density (*P*) in W cm^−2^ of the 5% Ni-SnS_2_@SnO_2_ electrode were calculated using eqn (3) and (4), respectively.3
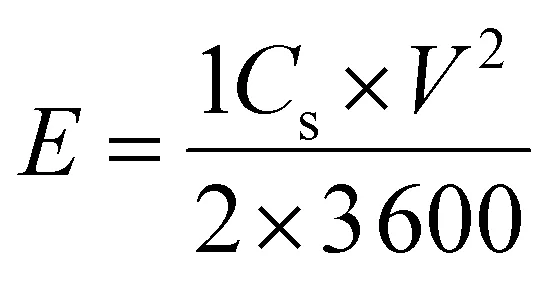
4
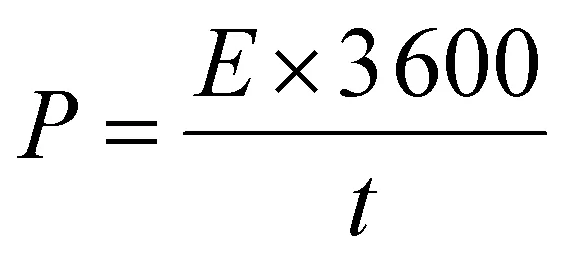
Here, *C*_s_ is the specific capacitance of the electrode (mF cm^−2^), *V* is the potential difference (V), and *t* is the discharge time (s).^2^

## 3 Results and discussion

### 3.1 Structural and morphological characterizations

The X-ray diffraction (XRD) patterns of the synthesized powders are presented in Fig. 2(a–c). The diffraction peaks observed at the 2*θ* values of 26.67°, 33.90°, 37.90°, 51.80°, and 54.70° are indexed to the (110), (101), (200), (211), and (220) crystal planes, respectively, of tetragonal rutile SnO_2_ (JCPDS no. 41-1445).^17,18^ In addition, the diffraction peaks located at the 2*θ* values of 15.11°, 28.32°, and 32.17° correspond to the (001), (100), and (101) planes of hexagonal SnS_2_, respectively (JCPDS no. 23-0677). No impurity phases were detected, confirming the high phase purity of the synthesized materials. Furthermore, no characteristic diffraction peaks associated with Ni were observed, indicating the successful incorporation of Ni ions into the SnS_2_ and SnO_2_ crystal lattices without forming secondary phases.^28,29^ For the SnS_2_@SnO_2_ sample, the broadened and weakened (001) diffraction peak at 15.11° suggests the suppressed growth of SnS_2_ nanosheets along the *c*-axis, resulting in a reduced crystallite thickness.^30^ The (001) peak shifts toward lower angles after Ni doping (from 15.11° to 14.98°), indicating an increase in the interlayer spacing of Ni-doped SnS_2_ compared with that of pristine SnS_2_, as shown in Fig. 2(b).

**Fig. 2:**
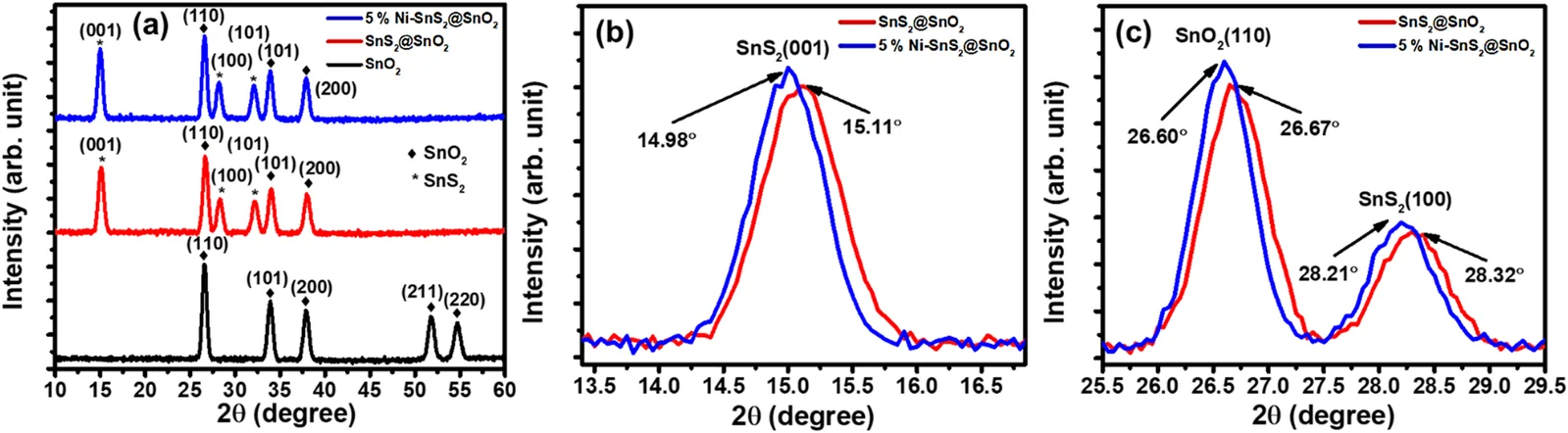
(a) XRD patterns of SnO_2_, SnS_2_@SnO_2_ and 5% Ni-doped SnS_2_@SnO_2_. (b) 2*θ* shifting of the SnS_2_ (001) diffraction plane and (c) 2*θ* shifting of the SnO_2_ (110) and SnS_2_ (100) diffraction planes due to Ni doping.

Similarly, in Fig. 2(c), the (100) peak of SnS_2_ and the (110) peak of SnO_2_ exhibit a shift toward lower angles. This expansion of the interlayer spacing and the formation of few-layered SnS_2_ may be attributed to the structural strain induced by lattice expansion upon Ni incorporation into the crystal lattice.^18^ Such structural modifications are highly beneficial for electrochemical performance. The enlarged interlayer spacing facilitates easier ion intercalation/deintercalation by reducing diffusion resistance and providing more accessible pathways for the electrolyte ions. The few-layered structure further improves the effective surface area and shortens the ion diffusion length, collectively leading to improved charge storage capability, faster reaction kinetics, and enhanced overall electrochemical performance.^29–31^

The surface morphologies of the as-synthesized materials were investigated using FESEM, as presented in Fig. 3(a–c). As shown in Fig. 3(a), SnO_2_ exhibits relatively non-uniform flower-like microspheres composed of interconnected nanoscale features. In contrast, SnS_2_@SnO_2_ (Fig. 3(b)) displays a more well-defined and uniform hierarchical flower-like spherical morphology, suggesting the self-assembly of nanoscale building blocks during the synthesis process. Notably, the Ni-SnS_2_@SnO_2_ composite (Fig. 3(c)) exhibits a distinct three-dimensional hierarchical architecture consisting of ultrathin, vertically oriented and interconnected nanoflower-like structures. Compared with the pristine SnO_2_ and SnS_2_@SnO_2_ samples, the Ni-incorporated composite presents a more homogeneous and densely interconnected flower-like morphology. Such a hierarchical 3D architecture can provide a highly accessible surface and facilitate effective electrolyte penetration into the electrode structure, thereby exposing a greater number of electrochemically accessible active sites. The interconnected nanoscale features may also provide favorable pathways for electrolyte-ion migration within the hierarchical structure, highlighting the structural advantage of the Ni-SnS_2_@SnO_2_ composite for electrochemical applications.

**Fig. 3:**
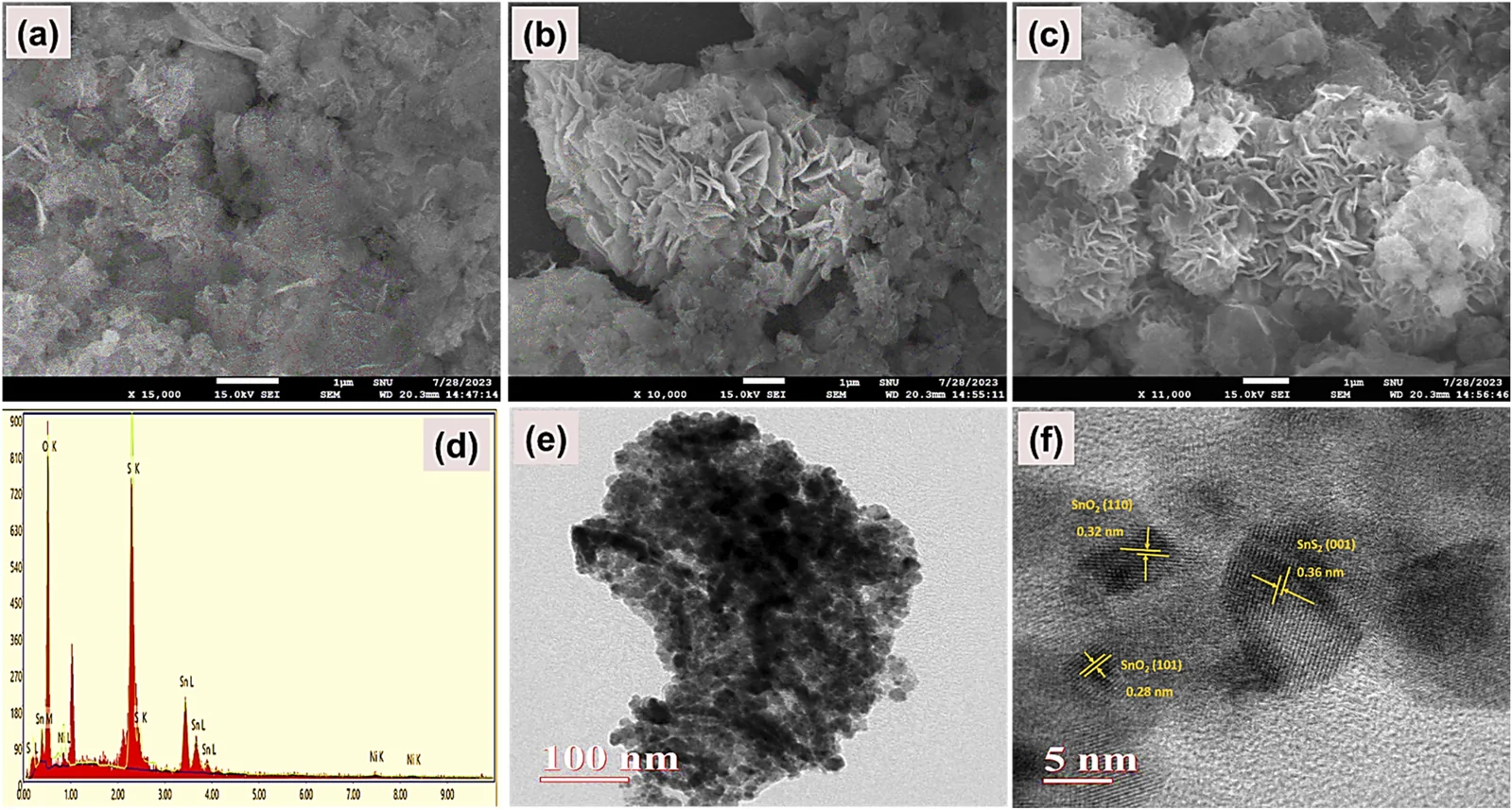
FESEM images of (a) SnO_2_, (b) SnS_2_@SnO_2_ and (c) 5% Ni-doped SnS_2_@SnO_2_. (d) EDX spectrum of 5% Ni-doped SnS_2_@SnO_2_. (e) TEM and (f) HRTEM images of 5% Ni-doped SnS_2_@SnO_2_.

EDX measurements demonstrate that Ni-SnS_2_@SnO_2_ consists of the Sn, O, S and Ni elements (Fig. 3(d)). The collective findings demonstrate that the Ni element has been successfully incorporated into the crystal structures of SnO_2_ and SnS_2_. This can be further confirmed by XRD measurements. The absence of any detectable impurity phases further confirms the high phase purity of the synthesized material. The detailed nanoscale morphology and microstructure of the as-synthesized Ni-SnS_2_@SnO_2_ nanocomposite were investigated using TEM and HRTEM. Fig. 3(e) shows the high-magnification TEM image of Ni-SnS_2_@SnO_2_ revealing its flower-like structure, consistent with the FESEM results. The high-resolution TEM image of Ni-SnS_2_@SnO_2_ (Fig. 3(f)) exhibits distinct lattice fringes, which confirm the well-crystallized structure of the SnS_2_ and SnO_2_ phases. The measured lattice spacings of 0.32 and 0.28 nm correspond to the (110) and (101) crystallographic planes of tetragonal SnO_2_, respectively. In addition, the interplanar spacing of 0.36 nm is indexed to the (001) plane of hexagonal SnS_2_, further confirming the coexistence of both crystalline phases in the nanocomposite. These findings for interlayer spacing confirm the successful formation of the Ni-SnS_2_@SnO_2_ nanocomposite.

The surface elemental composition and chemical states of the as-synthesized Ni-SnS_2_@SnO_2_ nanocomposite were analyzed by X-ray photoelectron spectroscopy (XPS). This analysis provides detailed insights into the valence states of the constituent elements as well as the surface chemical environment. The high-resolution XPS spectra shed light on the presence of the Sn, S, O, and Ni elements in the Ni-SnS_2_@SnO_2_ sample, as shown in Fig. 4(a–d).

**Fig. 4:**
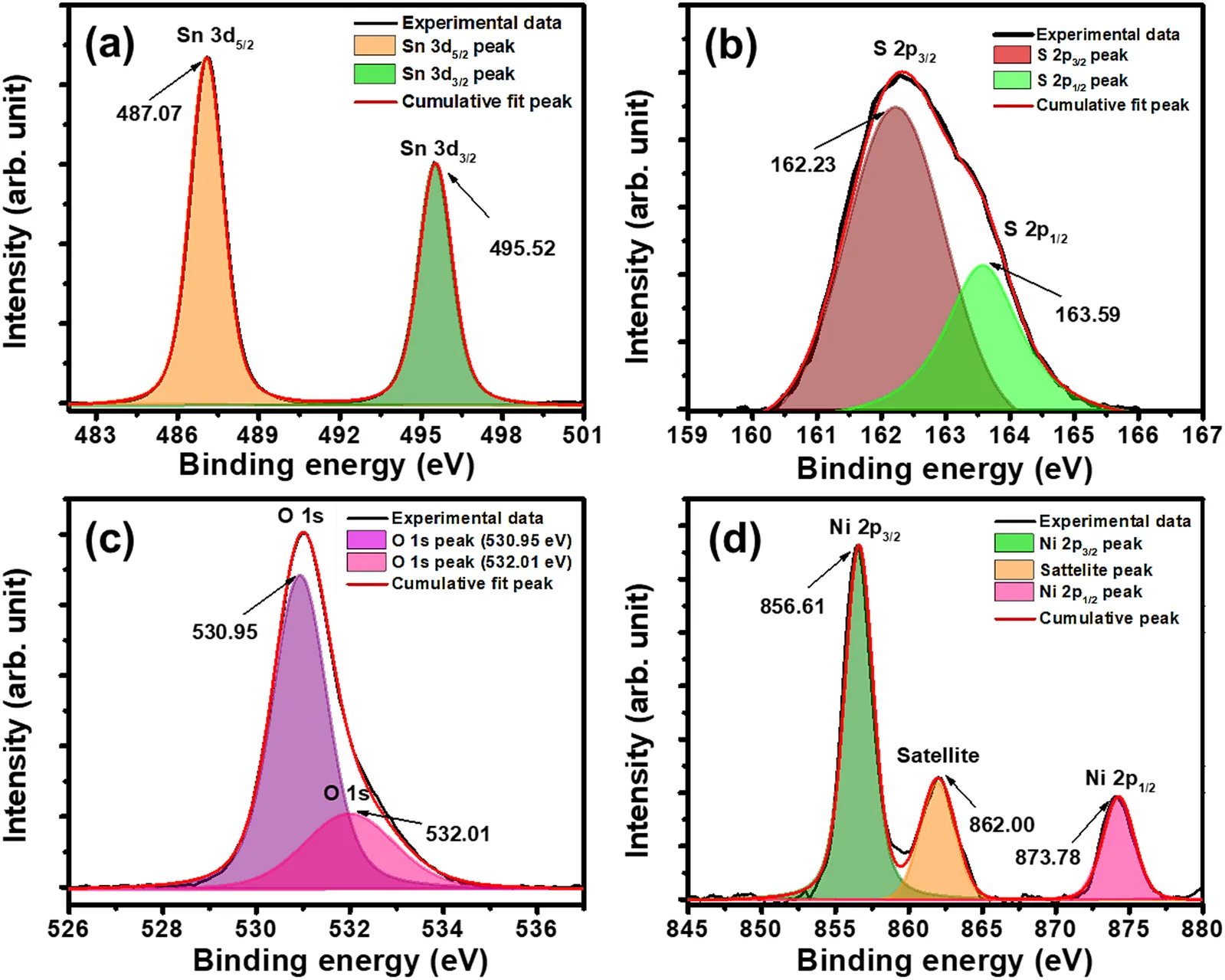
High-resolution XPS spectra of 5% Ni-doped SnS_2_@SnO_2_: (a) Sn 3d, (b) S 2p, (c) O 1s and (d) Ni 2p.

As shown in Fig. 4(a), the high-resolution Sn 3d XPS spectrum exhibits two characteristic peaks centered at the binding energies of 495.52 and 487.07 eV, corresponding to the photoelectron emissions of Sn 3d_3/2_ and Sn 3d_5/2_ spin–orbit components, respectively. The spin–orbit splitting between the two peaks is 8.45 eV, confirming the presence of Sn^4+^ in the prepared structure.^32,33^ Moving on to Fig. 4(b), the high-resolution XPS spectrum of S 2p reveals two peaks located at the binding energies of 162.23 and 163.59 eV, corresponding to S 2p_3/2_ and S 2p_1/2_ of S 2p, respectively, attributed to divalent S^2−^.^34^ The peaks occurring at 530.95 and 532.01 eV represent the O 1 s core-level that correspond to O^2−^ in the SnO_2_ lattice structure (Fig. 4(c)). Finally, the characteristic peaks located at 856.61 and 873.78 eV can be indexed to Ni 2p_3/2_ and Ni 2p_1/2_, respectively, as can be seen in Fig. 4(d). The Ni 2p_3/2_ and Ni 2p_1/2_ binding energies are distinctly different from those of NiS and NiS_2_, indicating the absence of secondary nickel sulfide phases. These observations confirm the successful substitutional incorporation of Ni into the SnS_2_ and SnO_2_ lattice frameworks rather than the formation of separate Ni-based compounds.^35–37^

### 3.2 Electrochemical investigations

The electrochemical capacitive behaviour of the SnO_2_, SnS_2_@SnO_2_, and Ni-SnS_2_@SnO_2_ electrodes was investigated in a 1 M Na_2_SO_4_ aqueous electrolyte using a standard three-electrode system, employing a platinum (Pt) counter electrode and an Ag/AgCl reference electrode. Fig. 5(a–c) illustrate the CV curves of the fabricated SnO_2_, SnS_2_@SnO_2_, and Ni-SnS_2_@SnO_2_ electrodes at various scan rates ranging from 10–100 mV s^−1^ within the potential range of 0.0 V to 0.8 V. All electrodes exhibit near-rectangular CV profiles, indicating a predominantly surface-controlled capacitive/pseudocapacitive charge-storage contribution, which facilitates rapid and reversible charge–discharge processes. It is observed that the current density response progressively rises with an increase in the scan rate, signifying the capacitive characteristics of the fabricated electrode. Furthermore, as the scan rate increases, the nature of the CV curves remains consistent, demonstrating favorable capacitance performance and efficient transport of the electrolyte ions to the electrode surface. Furthermore, the CV curve of Ni-SnS_2_@SnO_2_ displays a larger enclosed area, signifying its superior capacitive performance compared with the other two electrodes. Consequently, the morphology and optimal concentration of Ni in the Ni-SnS_2_@SnO_2_ nanocomposite significantly improves the electrochemical properties of the prepared electrode. Furthermore, the hierarchical flower-like architecture offers a high surface area with abundant electrochemically active sites, promoting efficient electrolyte ion diffusion and charge transfer at the electrode–electrolyte interface and thereby significantly enhancing the capacitive performance of the prepared electrode. Also, Ni doping in SnS_2_ and SnO_2_ leads to an expansion of the interlayer spacing, which facilitates easier ion intercalation and deintercalation. This structural modification reduces diffusion resistance and creates more accessible pathways for the electrolyte ions, thereby enhancing ion transport kinetics and improving the electrochemical performance. The specific capacitance values, calculated using eqn (1), were found to be 135.66, 129.83, 122.28, 117.25, 113.75 and 111.72 mF cm^−2^ for the synthesized Ni-SnS_2_@SnO_2_ nanocomposite at the scan rates of 10, 20, 40, 60, 80, and 100 mV s^−1^, respectively. In contrast, the specific capacitance value for pure SnO_2_ and SnS_2_@SnO_2_ are 49.15, 46.88, 43.06, 39.78, 35.98, and 35.24 mF cm^−2^ and 86.66, 85.21, 83.10, 80.31, 76.88, and 74.78, respectively, under the same scan rates (10 to 100 mV s^−1^). Fig. 5(d) represents the specific capacitance at various applied scan rates.

**Fig. 5:**
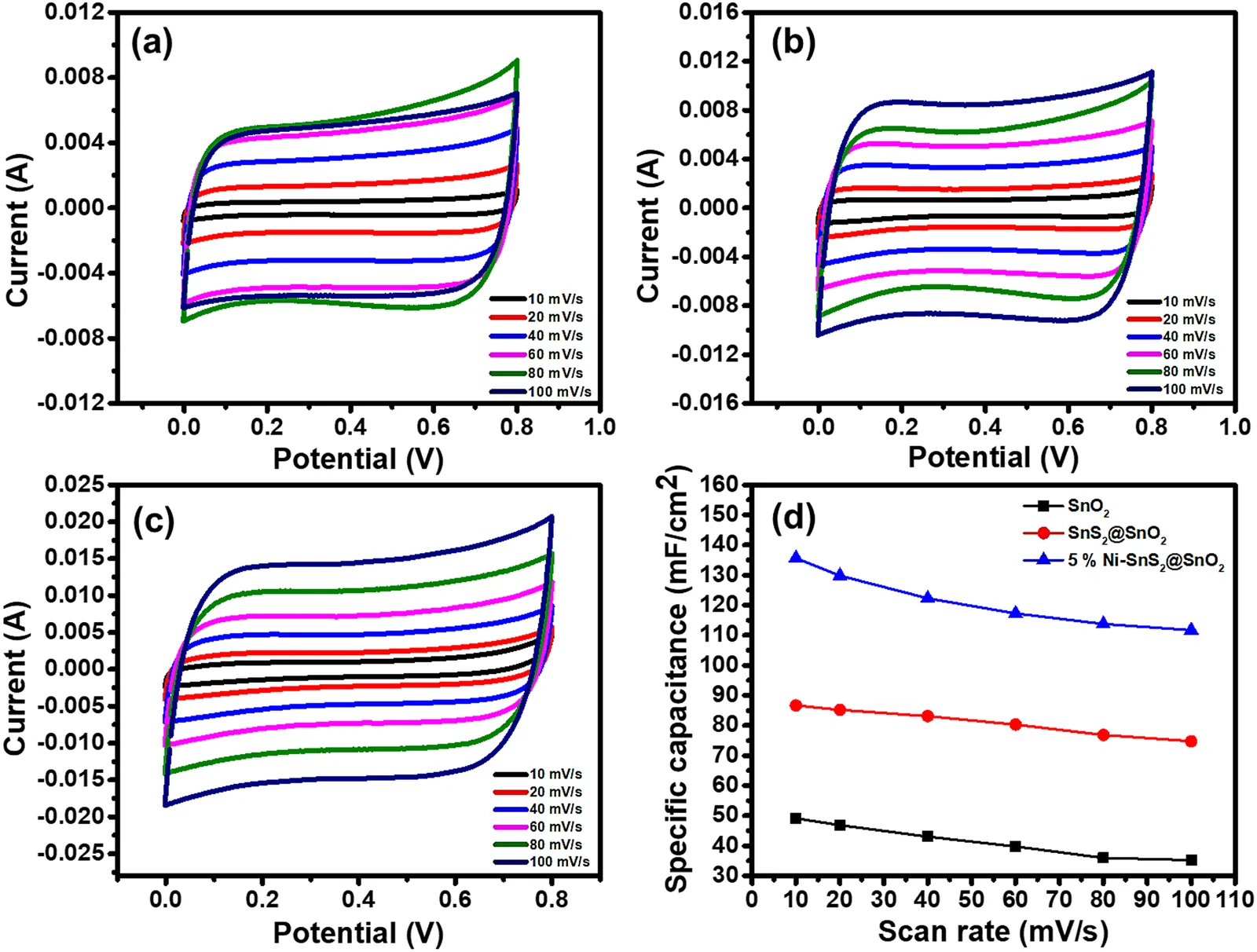
CV curves of (a) SnO_2_, (b) SnS_2_@SnO_2_ and (c) 5% Ni-doped SnS_2_@SnO_2_. (d) Specific capacitance *vs.* scan rate plots of all samples.

In the same context, the galvanostatic charge–discharge (GCD) measurements for the fabricated electrodes were performed in 1 M Na_2_SO_4_ within a similar potential range of 0.0 to 0.8 V (*vs.* Ag/AgCl) at various current densities from 0.5 mA cm^−2^ to 5 mA cm^−2^, as shown in Fig. 6(a–c). The nearly ideal triangular shape of the galvanostatic charge–discharge (GCD) curves, with insignificant deviation, confirms the excellent capacitive reversibility and predominant electric double-layer capacitive (EDLC) behavior of the prepared electrode, which align with the CV results. In this instance, eqn (2) was utilized to calculate the specific capacitance, yielding the values of 202.63, 149.89, 124.47, 105.03, and 98.37 mF cm^−2^ for Ni-SnS_2_@SnO_2_ at the current densities of 0.5, 1, 2, 3, and 5 mA cm^−2^, respectively. For comparison, electrochemical tests of pure SnO_2_ and SnS_2_@SnO_2_ were also conducted under the same parameters, as depicted in Fig. 6(a) and (b), respectively.

**Fig. 6:**
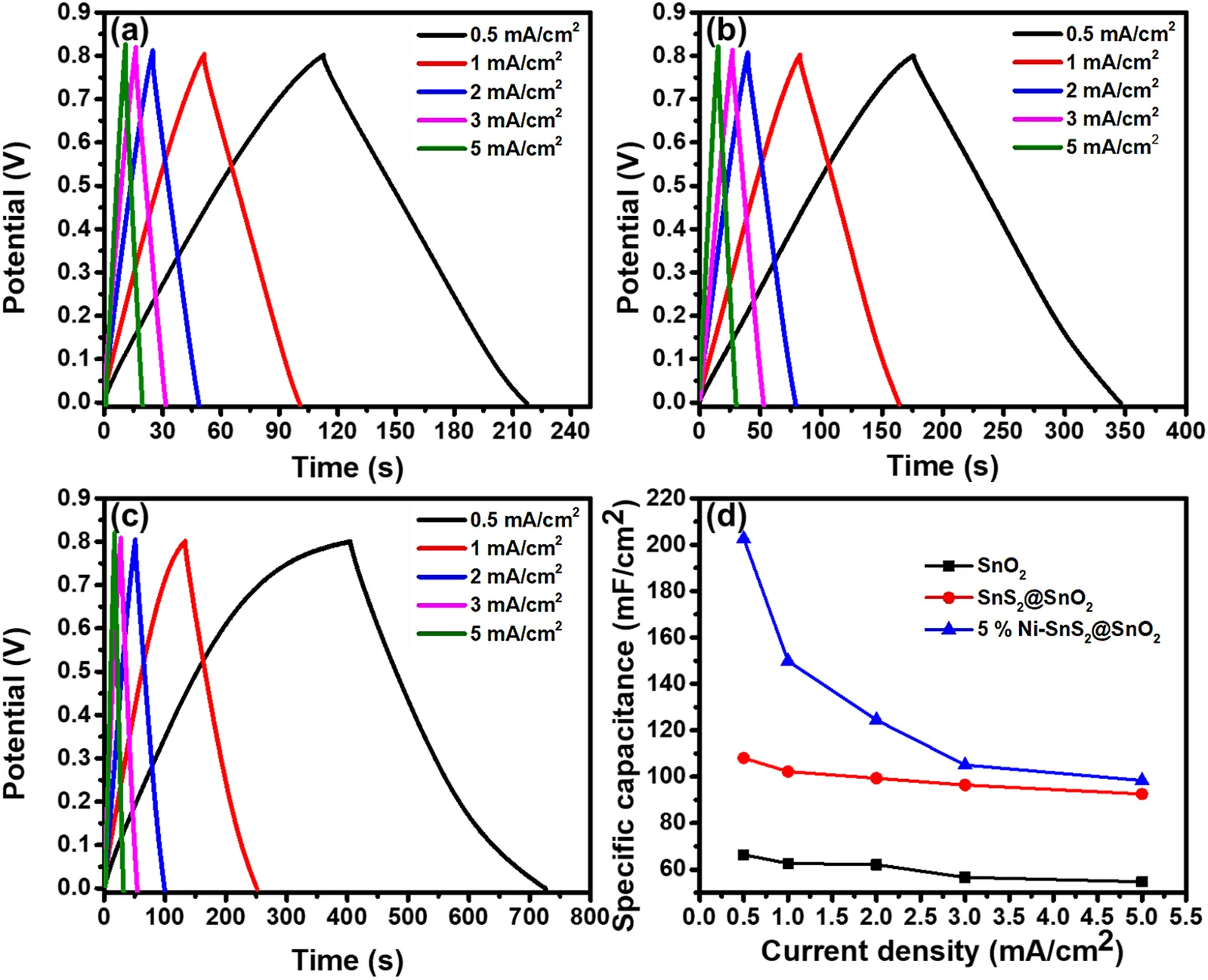
GCD curves of (a) SnO_2_, (b) SnS_2_@SnO_2_ and (c) 5% Ni-doped SnS_2_@SnO_2_. (d) Specific capacitance *vs.* current density plots of all samples.

The specific capacitance values obtained for these electrodes are 66.32, 62.63, 62.07, 56.62, and 54.70 mF cm^−2^ and 108.06, 102.17, 99.30, 96.45, and 92.56 mF cm^−2^ at current densities of 0.5, 1, 2, 3, and 5 mA cm^−2^, respectively. The maximum specific capacitance for the Ni-doped sample was recorded to be 202.63 mF cm^−2^ at the current density of 0.5 mA cm^−2^, while the other two electrodes exhibited values of only 66.32 and 108.06 mF cm^−2^ under the same parameters. Among the investigated electrodes, the Ni-SnS_2_@SnO_2_ nanocomposite exhibits the longest discharge time, reflecting its improved charge storage capability and resulting in the highest specific capacitance. Fig. 6(d) presents the variation in the specific capacitance of the prepared electrodes as a function of the current density. It is evident that the specific capacitance diminishes as the current density increases (from 0.5 to 5.0 mA cm^−2^). This behavior is attributed to the restricted diffusion of electrolyte ions into the interior of the electrode at higher current densities, which limits access to electrochemically active sites and consequently reduces the specific capacitance.

To gain further insights into the electrochemical charge storage kinetics, CV measurements were conducted at various scan rates, and the resulting voltammograms were analysed. The contributions of capacitive and diffusion-controlled processes were evaluated through power-law analysis and quantitative separation of the current response. The electrochemical kinetics of the electrodes were evaluated by analysing the relationship between the peak current (*i*) and scan rate (*ν*), which follows the power-law equation *i* = *aν*^*b*^, where *a* and *b* are adjustable constants. By transforming this equation into its logarithmic form, log(*i*) = log(*a*) + *b* log(*ν*), the *b*-value is obtained from the slope of the linear fit of log(*i*) *versus* log(*ν*), as illustrated in Fig. 7(a–c) for the anodic reaction and Fig. 7(d)–(f) for the cathodic reaction. The *b*-value provides valuable insights into the charge storage mechanism. A *b*-value approaching 0.5 indicates a diffusion-controlled faradaic process, whereas a *b*-value close to 1.0 signifies a surface-controlled capacitive process.^38–44^ The calculated *b* values for the anodic and cathodic reactions of the SnO_2_, SnS_2_@SnO_2_, and 5% Ni-SnS_2_@SnO_2_ electrodes are shown in Fig. 7(a–f), and these values are summarised in Table 1.

**Fig. 7:**
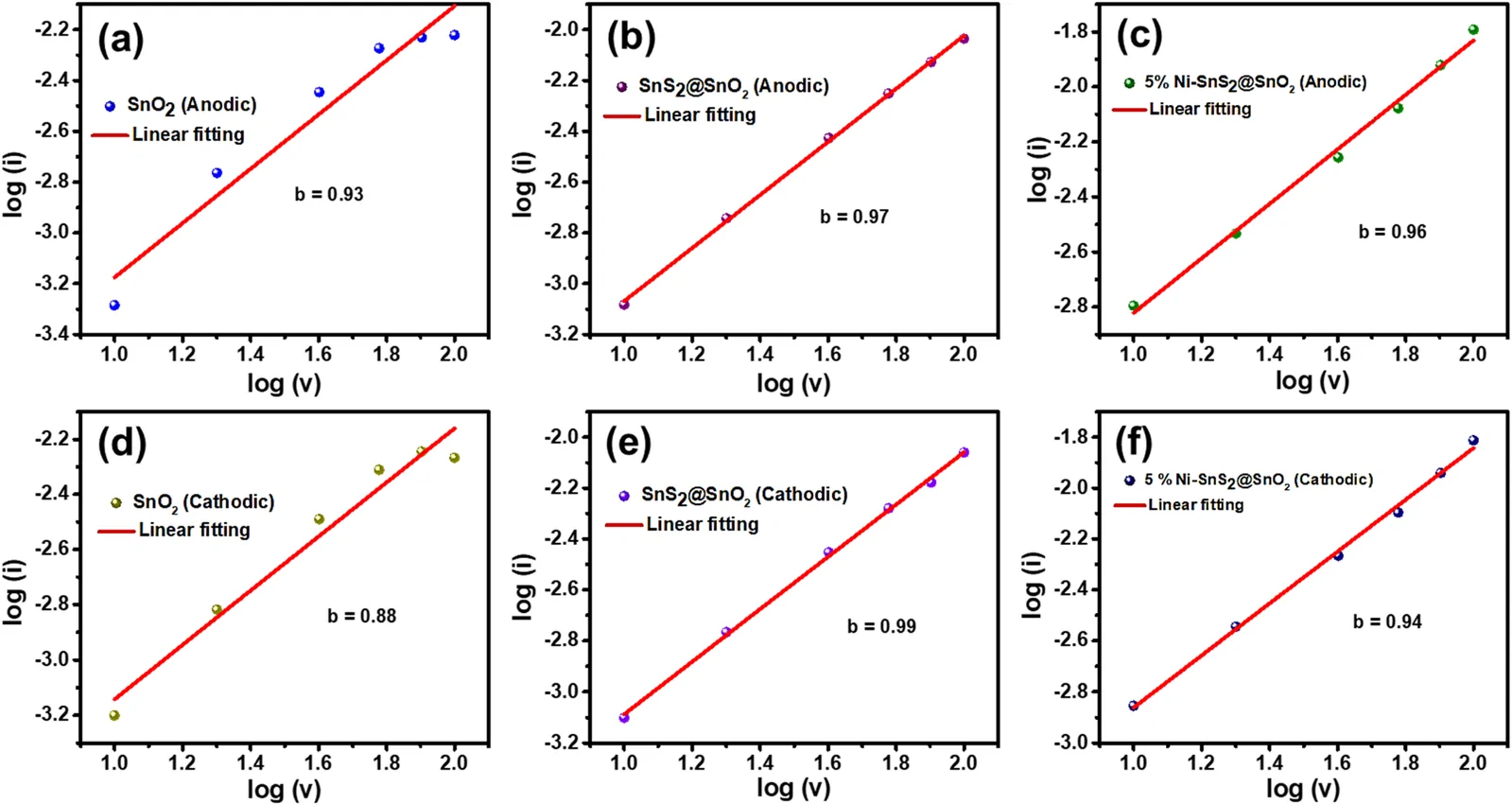
Calculated *b* values for the anodic reactions of (a) SnO_2_, (b) SnS_2_@SnO_2_ and (c) 5% Ni-SnS_2_@SnO_2_. Calculated *b* values for the cathodic reactions of (d) SnO_2_, (e) SnS_2_@SnO_2_ and (f) 5% Ni-SnS_2_@SnO_2_.

**Table 1:** Summary of the *b* values and capacitive and diffusion-controlled contributions for the anodic and cathodic reactions of SnO_2_, SnS_2_@SnO_2_ and 5% Ni-SnS_2_@SnO_2_ at different scan rates

Electrode material	Anodic reaction			Cathodic reaction		
	*b* value	Capacitive (%)	Diffusive (%)	*b* value	Capacitive (%)	Diffusive (%)
SnO_2_	0.93	77.78%@10 mV s^−1^	22.22%@10 mV s^−1^	0.88	68.90%@10 mV s^−1^	31.10%@10 mV s^−1^
		91.72%@100 mV s^−1^	8.28%@100 mV s^−1^		87.50%@100 mV s^−1^	12.50%@100 mV s^−1^
SnS_2_@SnO_2_	0.97	90.77%@10 mV s^−1^	9.23%@10 mV s^−1^	0.99	94.67%@10 mV s^−1^	5.33%@10 mV s^−1^
		96.88%@100 mV s^−1^	3.12%@100 mV s^−1^		98.25%@100 mV s^−1^	1.75%@100 mV s^−1^
5% Ni-SnS_2_@SnO_2_	0.96	90.58%@10 mV s^−1^	9.42%@10 mV s^−1^	0.94	86.95%@10 mV s^−1^	13.05%@10 mV s^−1^
		96.82%@100 mV s^−1^	3.18%@100 mV s^−1^		95.47%@100 mV s^−1^	4.53%@100 mV s^−1^

For the pristine SnO_2_ electrode (Fig. 7(a) and (d)), the *b* values are closer to the predominantly surface-controlled regime, indicating that the electrochemical charge storage originates from both ion diffusion and surface control within the surface and bulk structure of the electrode. This behaviour is typical for metal oxide electrodes, where the electrochemical reaction is governed by the insertion/extraction of the electrolyte ions. For the SnS_2_@SnO_2_ heterostructure electrode, the *b* values increase compared with that of pristine SnO_2_, suggesting the emergence of additional surface-controlled charge storage, as can be seen in Fig. 7(b) and (e). The formation of the heterostructure may introduce a larger electrochemically active surface area and improve electronic conductivity, promoting faster charge-transfer kinetics at the electrode–electrolyte interface. As observed in Fig. 7(c) and (f), the 5% Ni-SnS_2_@SnO_2_ electrode shows a further modification in the *b* values, indicating a change in the contribution from more capacitive processes to apparently diffusive. The incorporation of Ni ions into the SnS_2_@SnO_2_ framework likely introduces additional redox-active sites and improves charge transport within the electrode by modifying the inter-planar spacing, which may help in ion intercalation processes. As a result, the electrode exhibits improved electrochemical kinetics with a mixed charge storage behaviour.

To quantitatively differentiate the capacitive and diffusion-controlled contributions to charge storage, the current response at a given potential can be expressed as *i*(*v*) = *k*_1_*ν* + *k*_2_*ν*^1/2^, where *k*_1_*ν* represents surface-controlled capacitive processes and *k*_2_*ν*^1/2^ corresponds to diffusion-limited faradaic contributions.^38–44^ The variation in capacitive and diffusion contributions for the anodic and cathodic reactions with the scan rate is presented in Fig. 8(a)–(f), and the corresponding values are summarized in Table 1.

**Fig. 8:**
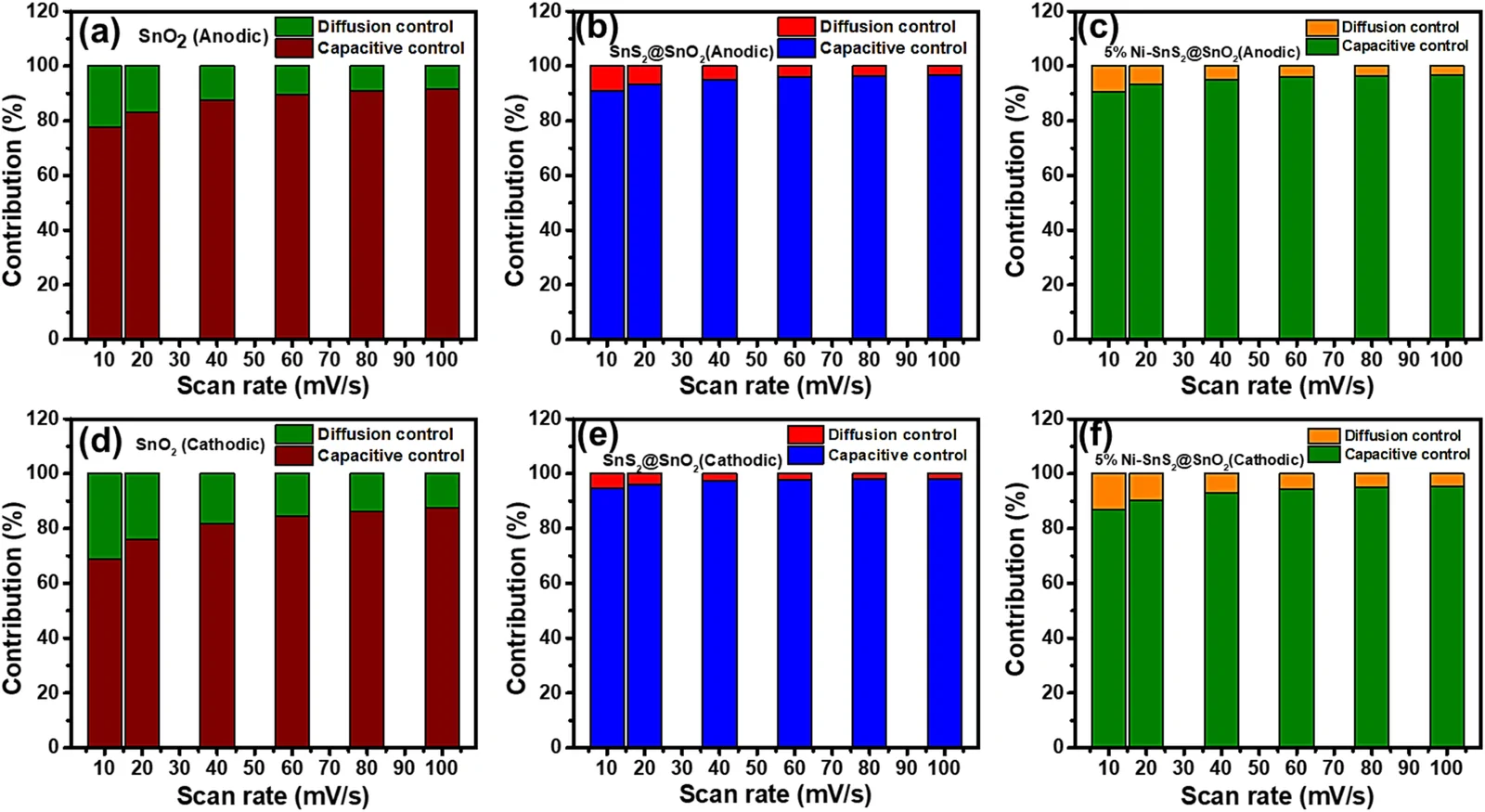
Diffusive and capacitive control contribution values for the anodic reaction of (a) SnO_2_, (b) SnS_2_@SnO_2_ and (c) 5% Ni-SnS_2_@SnO_2_ at different scan rates. Diffusive and capacitive control contribution values for the cathodic reaction of (d) SnO_2_, (e) SnS_2_@SnO_2_ and (f) 5% Ni-SnS_2_@SnO_2_ at different scan rates.

As can be seen in Fig. 8(a) and (d), for the SnO_2_ electrode, the predominantly surface-controlled contribution at most scan rates indicates that the charge storage process is governed by ion diffusion and surface storage within the electrode material. The capacitive contribution increases progressively with increasing scan rate because the reduced diffusion time limits ion transport into the bulk of the electrode, thereby enhancing the relative contribution of surface-controlled charge storage. In contrast, the SnS_2_@SnO_2_ electrode exhibits a noticeable increase in the capacitive contribution with increasing scan rates (Fig. 8(b) and (e)). The presence of the SnS_2_ component enhances electrical conductivity and provides additional electrochemically active sites. This structural modification facilitates faster electron transport and improves electrolyte accessibility, thereby enhancing the surface-controlled charge storage process.

The 5% Ni-SnS_2_@SnO_2_ electrode demonstrates an enhancement in the diffusive contribution compared with the SnS_2_@SnO_2_ electrode, as shown in Fig. 8(c) and (f). To further evaluate the contribution of capacitive processes, the capacitive and diffusion-controlled current distributions were analysed at a representative scan rate of 10 mV s^−1^, as shown in Fig. 9 and 10.

**Fig. 9:**
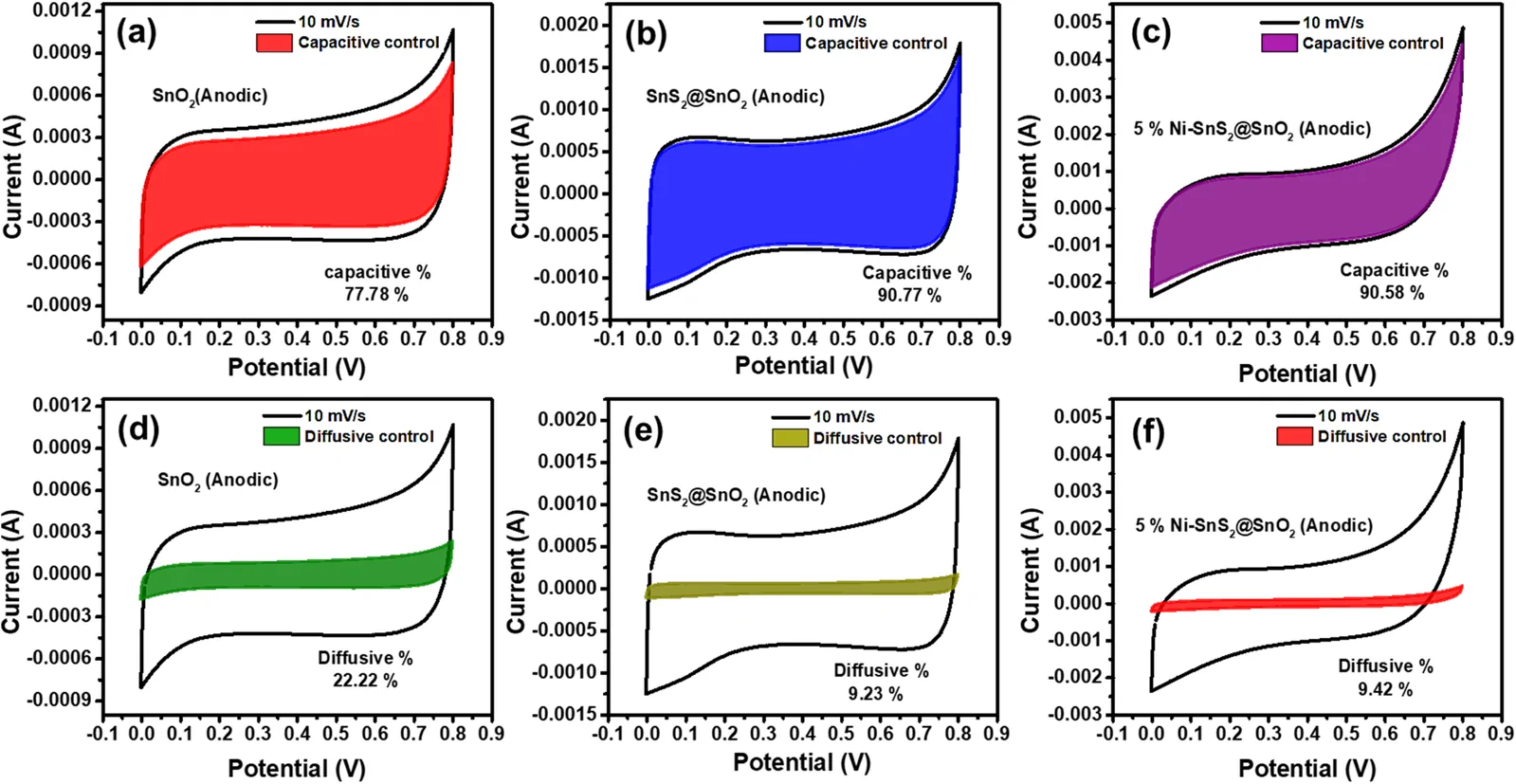
Capacitive control contribution value for the anodic reaction of (a) SnO_2_, (b) SnS_2_@SnO_2_ and (c) 5% Ni-SnS_2_@SnO_2_ at a scan rate of 10 mV s^−1^. Diffusive control contribution value for the anodic reaction of (d) SnO_2_, (e) SnS_2_@SnO_2_ and (f) 5% Ni-SnS_2_@SnO_2_ at a scan rate of 10 mV s^−1^.

**Fig. 10:**
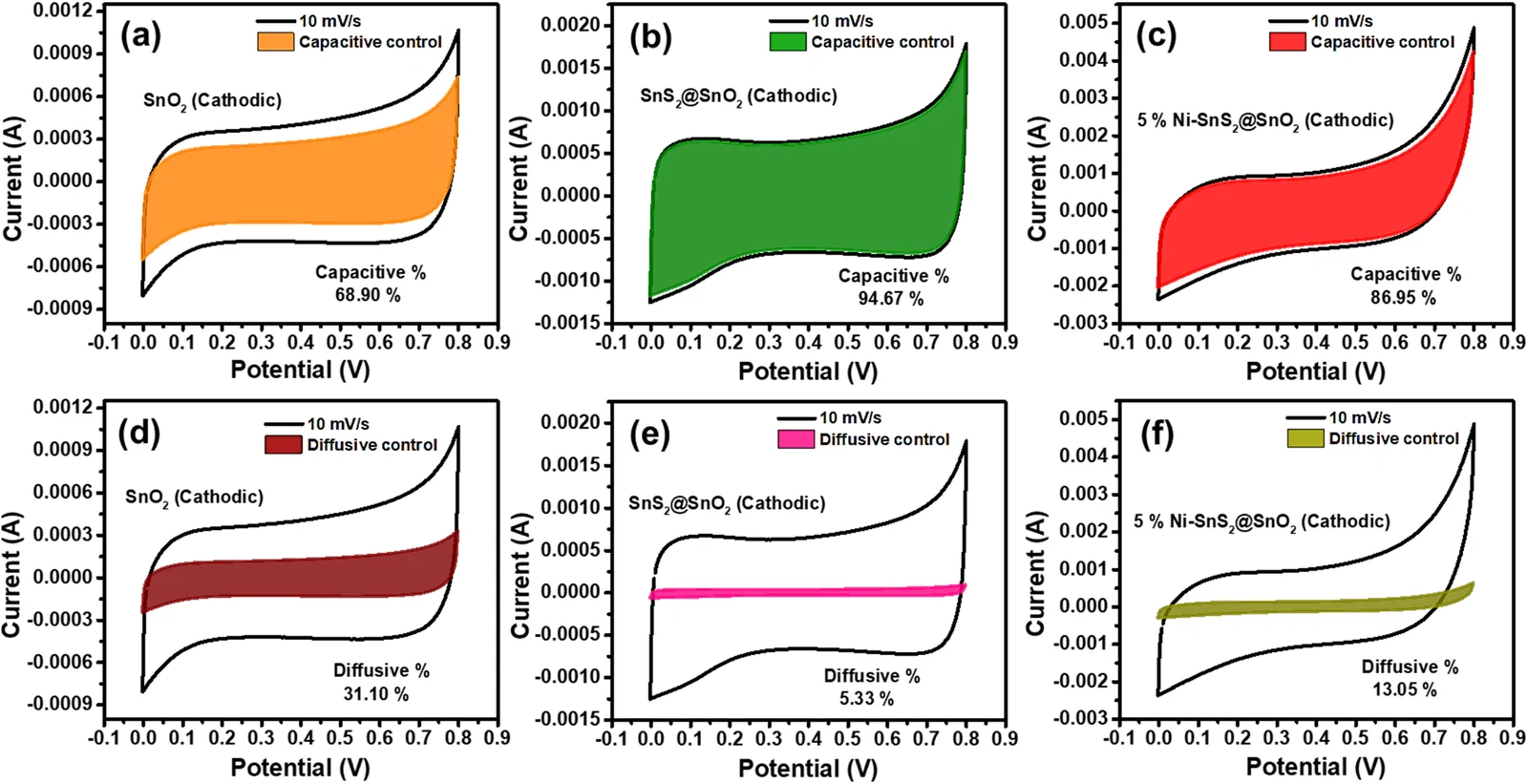
Capacitive control contribution value for the cathodic reaction of (a) SnO_2_, (b) SnS_2_@SnO_2_ and (c) 5% Ni-SnS_2_@SnO_2_ at a scan rate of 10 mV s^−1^. Diffusive control contribution value for the cathodic reaction of (d) SnO_2_, (e) SnS_2_@SnO_2_ and (f) 5% Ni-SnS_2_@SnO_2_ at a scan rate of 10 mV s^−1^.

Fig. 9(a) and (d) for the anodic reaction, and Fig. 10(a) and (d) for the cathodic reaction show that the predominantly surface-controlled component remains dominant across the potential window for the SnO_2_ electrode. For the SnS_2_@SnO_2_ electrode (Fig. 9(b), (e) and 10(b), (e)), the capacitive contribution becomes more significant across the potential range. The heterostructure formation between SnS_2_ and SnO_2_ provides a synergistic effect that enhances electron transport and exposes additional active sites for electrochemical reactions. The 5% Ni-SnS_2_@SnO_2_ electrode (Fig. 9(c), (f) and 10(c), (f)) exhibits the moderate capacitive-diffusive contribution among the three electrodes. This is because Ni doping leads to an increase in interlayer spacing that facilitates easier ion intercalation/deintercalation by reducing diffusion resistance and providing more accessible pathways for electrolyte ions (as confirmed by the XRD results). Based on the combined analysis of *b* values and capacitive-diffusion contributions, the charge storage mechanism of the electrodes evolves significantly with structural modification *via* composite formation and doping.

Therefore, the improved electrochemical kinetics observed in the 5% Ni-SnS_2_@SnO_2_ electrode can be attributed to the synergistic effects of heterostructure engineering and transition metal doping, which collectively enhance surface redox reactions and facilitate efficient ion diffusion.

In the three-electrode system, the Ni-doped SnS_2_@SnO_2_ electrode undergoes a reversible interaction with Na^+^ ions from the 1 M Na_2_SO_4_ electrolyte. Charge storage might involve a combination of Na^+^ adsorption at the electrode/electrolyte interface, surface redox reactions, and partial Na^+^ intercalation/deintercalation within the SnS_2_@SnO_2_ heterostructure. The Sn–S/Sn–O active sites provide redox-active centres, while the SnS_2_/SnO_2_ heterointerface and Ni incorporation may facilitate electron transport and improve the Na^+^ accessibility. These synergistic processes contribute to the enhanced specific capacitance and rapid charge-storage kinetics of the Ni-doped SnS_2_@SnO_2_ electrode.

To achieve a more profound comprehension of the capacitive properties of the fabricated SnO_2_, SnS_2_@SnO_2_, and Ni-SnS_2_@SnO_2_ electrodes, impedance measurements were conducted under open-circuit potential conditions over a frequency range of 0.01 Hz to 10^5^ Hz. The corresponding Nyquist plots are presented in Fig. 11(a). The Nyquist plots exhibit a predominantly inclined response without a well-defined semicircular arc; therefore, the reliable quantitative determination of the charge-transfer resistance (*R*_ct_) is not appropriate. Instead, the solution/ohmic resistance (*R*_s_), obtained from the high-frequency intercept on the real axis, was considered for comparison. The *R*_s_ values of SnO_2_, SnS_2_@SnO_2_, and 5% Ni-SnS_2_@SnO_2_ are 6.16, 5.41, and 3.83 Ω, respectively. The EIS results provide complementary evidence for the changes observed in the CV-derived *b* values. Pristine SnO_2_ exhibits a relatively higher *R*_s_ value, consistent with less favourable overall charge transport and its mixed surface-controlled/diffusion-influenced charge-storage behaviour. The formation of the SnS_2_@SnO_2_ heterostructure decreases *R*_s_, indicating improved ohmic charge transport through the heterointerface, which is consistent with the increased *b* value and the resulting greater contribution of surface-controlled processes. For 5% Ni-SnS_2_@SnO_2_, further variations in *R*_s_, together with the modified *b* value, indicate that Ni incorporation alters the overall charge-/ion-transport characteristics of the electrode. The Ni-induced lattice modification and increased interlayer spacing can facilitate Na^+^ accessibility and transport within the heterostructure, thereby increasing the diffusion-influenced contribution. Thus, the *R*_s_ and *b*-value results collectively indicate that heterostructure formation and Ni incorporation modify both charge transport and ion-storage kinetics, leading to a mixed contribution from surface-controlled and diffusion-influenced processes.^44^

**Fig. 11:**
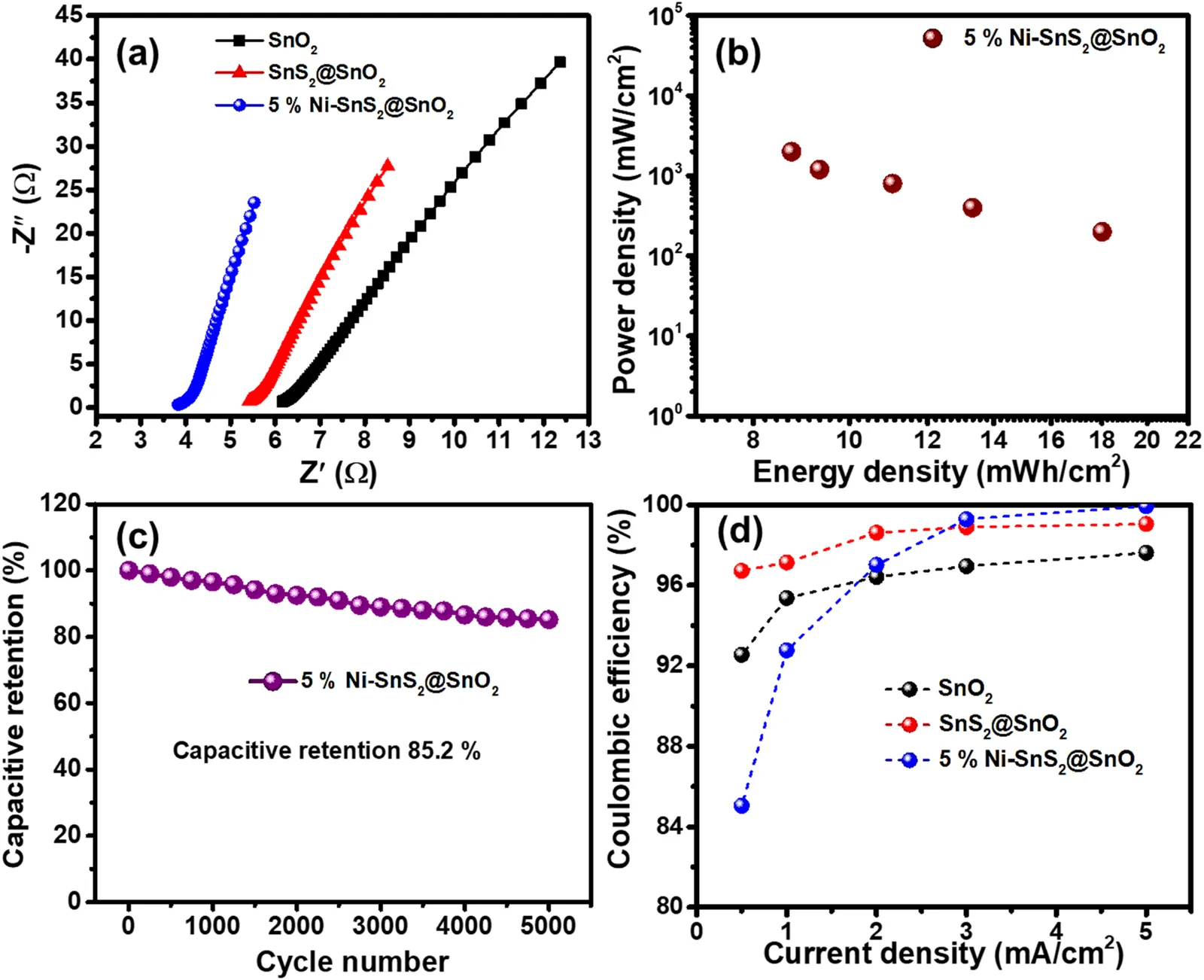
(a) Nyquist plots of SnO_2_, SnS_2_@SnO_2_ and 5% Ni-doped SnS_2_@SnO_2_. (b) Ragone plot of 5% Ni-doped SnS_2_@SnO_2_. (c) Cyclic stability of 5% Ni-doped SnS_2_@SnO_2_. (d) Estimated coulombic efficiency (CE%) of SnO_2_, SnS_2_@SnO_2_ and 5% Ni-doped SnS_2_@SnO_2_.

Based on the GCD measurements, the energy density and power density were determined using eqn (3) and (4), respectively. The relationship between power and energy densities for the fabricated Ni-SnS_2_@SnO_2_ electrode was analyzed by constructing a Ragone plot (Fig. 11(b)). Energy densities were calculated to be 18.01, 13.32, 11.06, 9.33, and 8.74 mW h cm^−2^, while power densities were 199.98, 399.89, 799.67, 1199.14, and 1998.89 mW cm^−2^ at 0.5, 1, 2, 3, and 5 mA cm^−2^ current densities, respectively, for Ni-SnS_2_@SnO_2_. The maximum energy density of Ni-SnS_2_@SnO_2_ was found to be 18.01 mW h cm^−2^, while the corresponding power density was 199.98 mW cm^−2^ at the current density of 0.5 mA cm^−2^.

The cycling stability of the Ni-SnS_2_@SnO_2_ electrode was systematically evaluated by continuous galvanostatic charge–discharge cycling at a constant current density of 1 mA cm^−2^ to examine its long-term electrochemical durability (Fig. 11(c)). The fabricated Ni-SnS_2_@SnO_2_ electrode retains 85.2% of its initial capacitance even after 5000 consecutive charge and discharge cycles. It can be concluded that the flower-like structure of the fabricated Ni-SnS_2_@SnO_2_ offers abundant electroactive sites, thereby facilitating electron transfer and ion migration.

The coulombic efficiency (CE) of the electrodes was evaluated to assess the reversibility of the charge–discharge process and the efficiency of electron/ion transport during electrochemical cycling, as shown in Fig. 11(d). Coulombic efficiency is defined as the ratio of the discharge time to the charge time and reflects the extent of charge recovery in each cycle. It can be expressed by the equation 
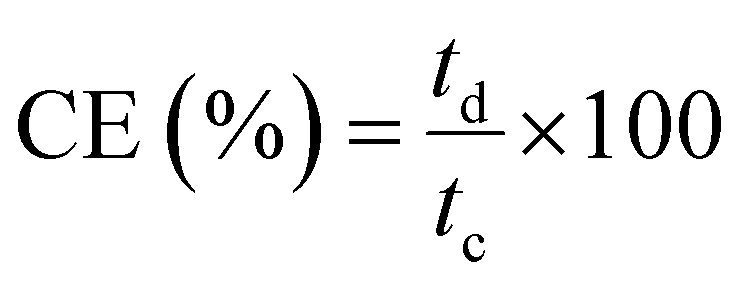
, where, *t*_d_ and *t*_c_ are the discharge time and charging time, respectively. The SnO_2_ electrode exhibits a relatively lower coulombic efficiency (CE% ranging from 92.55 to 97.62), particularly at higher current densities. This behavior can be attributed to its diffusion-controlled charge storage mechanism, as previously confirmed by charge storage kinetics with relatively smaller *b*-values. In diffusion-controlled systems, ion insertion/extraction into the bulk structure is often associated with higher polarization and irreversible losses, leading to reduced coulombic efficiency. In contrast, the SnS_2_@SnO_2_ heterostructure electrode demonstrates significantly improved coulombic efficiency (CE% ranging from 96.72 to 99.05). This enhancement is consistent with the earlier observation of increased *b*-values and a higher capacitive contribution. The improved efficiency arises from the synergistic effect of the heterostructure, which enhances the electrical conductivity and provides a larger electrochemically active surface area. As a result, surface-controlled redox reactions become more dominant, enabling faster and more reversible charge storage with minimal energy loss. The 5% Ni-SnS_2_@SnO_2_ electrode exhibits the highest and most stable coulombic efficiency among all samples (CE% ranging from 85.05 to 99.93). This improvement correlates well with its mixed capacitive-diffusive charge storage behavior observed from both *b*-value analysis and quantitative contribution studies. The incorporation of Ni introduces additional redox-active sites and improves electronic pathways while modifying the interlayer spacing to facilitate ion diffusion. This dual enhancement ensures efficient charge transfer and reduces irreversible side reactions, thereby improving the coulombic efficiency. Furthermore, the relatively stable coulombic efficiency at increasing current densities for all electrodes indicates their superior rate capability and electrochemical reversibility.^45,46^

Finally, we compared the electrochemical behaviour of various SnO_2_-based materials utilizing different electrolytes for supercapacitor applications with that of the Ni-SnS_2_@SnO_2_ electrode developed in the present work. It is observed that the specific capacitance of Ni-SnS_2_@SnO_2_ is higher than the previously reported values, as can be seen in Table 2.

**Table 2:** Comparison of the electrochemical behaviour of various SnO_2_-based materials utilizing different electrolytes for supercapacitor applications with that of the Ni-SnS_2_@SnO_2_ electrode

Materials	Specific capacitance	Current density or scan rate	Electrolyte	Capacity retention (%)	Cycles	Ref.
SnO_2_ nanoparticles	14 F g^−1^	1 A g^−1^	2 M KOH	—	—	14
MoS_2_-SnO_2_	61.6 F g^−1^	1 A g^−1^	2 M KOH	90	1000	14
SnO_2_ QDs	10.0 F g^−1^	20 mV s^−1^	0.5 M KOH	91	1000	15
SO_4_^2−^/SnO_2_	51.0 F g^−1^	5 mV s^−1^	3 M H_2_SO_4_	—	2000	16
SnO_2_/ACW	30.46 F g^−1^	10 mV s^−1^	1 M Na_2_SO_4_	108.9%	6000	22
SnO_2_/graphene	34.6 F g^−1^	1 V s^−1^	1 M H_2_SO_4_	—	—	23
SnO_2_/graphene	42.7 F g^−1^	50 mV s^−1^	2 M KCl	62	100	24
Nanocrystalline SnO_2_	66 F g^−1^	10 mV s^−1^	0.5 M Na_2_SO_4_	—	—	25
SnO_2_ nanospheres	25.8 F g^−1^	10 mV s^−1^	1 M KOH	—	—	26
RGO/SnO_2_	0.37 F cm^−2^	1 mA cm^−2^	2 M KOH	80.4	1000	27
SnO_2_	66.32 mF cm^−2^ (24.56 F g^−1^)					This work
SnS_2_@SnO_2_	108.06 mF cm^−2^ (40.02 F g^−1^)	0.5 mA cm^−2^	1 M Na_2_SO_4_			
Ni-SnS_2_@SnO_2_	202.63 mF cm^−2^ (75.05 F g^−1^)			85.2%	5000	

## 4 Conclusions

To summarize, a hierarchical Ni-SnS_2_@SnO_2_ nanocomposite was successfully synthesized *via* a facile solvothermal method, and its performance as a supercapacitor electrode was evaluated in an aqueous electrolyte using a three-electrode configuration. Structural analysis indicates that Ni doping induces lattice expansion, increases interlayer spacing, enhances ion accessibility and facilitates faster ion intercalation/deintercalation. Electrochemical studies reveal that the Ni-SnS_2_@SnO_2_ electrode exhibits improved charge storage kinetics with a mixed capacitive-diffusion-controlled mechanism, as supported by CV-based kinetic analysis. The synergistic effects of heterostructure formation and Ni incorporation enhance electrical conductivity, introduce additional redox-active sites, and optimize ion transport pathways. EIS results further confirm the reduced solution resistance and enhanced electrolyte ion diffusion kinetics of the Ni-SnS_2_@SnO_2_ electrode, demonstrating improved charge transport and faster electrochemical reaction kinetics. As a result, the electrode delivers a high areal capacitance of 202.63 mF cm^−2^ at 0.5 mA cm^−2^, along with excellent rate capability and good electrochemical stability. It retains ∼85% of its capacitance after 5000 charge–discharge cycles. The enhanced coulombic efficiency (85.05% to ∼99.93%) of the 5% Ni-SnS_2_@SnO_2_ electrode confirms its improved electrochemical reversibility and superior rate capability. Overall, the enhanced performance is attributed to the combined structural and compositional advantages, demonstrating that Ni-SnS_2_@SnO_2_ is a promising electrode material for advanced supercapacitors and energy storage applications.

## Author contributions

Ravindra Kumar: conceptualization, data curation, investigation, methodology, writing – original draft. Diksha Sharma: formal analysis, visualization. Geetika Patel: formal analysis, visualization. Gurupada Maity: conceptualization, data curation, investigation, formal analysis, validation, writing – review and editing. Ashish Kumar Keshari: conceptualization, methodology, resources, supervision, writing – review and editing.

## Conflicts of interest

There are no conflicts to declare.

## Data Availability

The experimental data that support the findings of this study are available from the corresponding authors [Dr Ashish Kumar Keshari and Dr Gurupada Maity] upon reasonable request.
